# Effects of Endurance Training Intensity on Pulmonary Diffusing Capacity at Rest and after Maximal Aerobic Exercise in Young Athletes

**DOI:** 10.3390/ijerph182312359

**Published:** 2021-11-24

**Authors:** Rim Dridi, Nadia Dridi, Karuppasamy Govindasamy, Nabil Gmada, Ridha Aouadi, Hervé Guénard, Ismail Laher, Ayoub Saeidi, Katsuhiko Suzuki, Anthony C. Hackney, Hassane Zouhal

**Affiliations:** 1 Research Unit 17JS01 (Sport, Performance, Health and Society) Higher Institute of Sport and Physical Education of Ksar Said, University of La Manouba, Tunis 2010, Tunisia; dridirim80@yahoo.fr (R.D.); nadiadridi@gmail.com (N.D.); ridhaaouadi@gmail.com (R.A.); 2 Department of Physical Education & Sports Science, SRM Institute of Science and Technology, Kattankulathur 603203, Tamilnadu, India; gowthamadnivog@gmail.com; 3 Physical Education and Sport Sciences Department, Sultan Qaboos University, Muscat 123, Oman; ngmada@yahoo.fr; 4 Department of Physiology, Victor Segalen University, 33076 Bordeaux, France; guenardherve@hotmail.com; 5 Department of Anesthesiology, Pharmacology and Therapeutics, Faculty of Medicine, University of British Columbia, Vancouver, BC V6T 1Z3, Canada; ismail.laher@ubc.ca; 6 Department of Physical Education and Sport Sciences, University of Kurdistan, Pasdaran St., Sanandaj 6617715175, Iran; saeidi_as68@yahoo.com; 7 Faculty of Sport Sciences, Waseda University, Tokorozawa 359-1192, Japan; 8 Department of Exercise & Sport Science, University of North Carolina, Chapel Hill, NC 27599, USA; thackney@med.unc.edu; 9 M2S (Laboratoire Mouvement, Sport, Santé)—EA 1274, Université de Rennes, 35000 Rennes, France; 10 Institut International des Sciences du Sport (2I2S), 35850 Irodouer, France

**Keywords:** aerobic training, pulmonary capillary blood volume, alveolar–capillary membrane, alveolar volume, NO/CO transfer

## Abstract

This study compared the effects of varying aerobic training programs on pulmonary diffusing capacity (TL_CO_), pulmonary diffusing capacity for nitric oxide (TL_NO_), lung capillary blood volume (Vc) and alveolar–capillary membrane diffusing capacity (DM) of gases at rest and just after maximal exercise in young athletes. Sixteen healthy young runners (16–18 years) were randomly assigned to an intense endurance training program (IET, *n* = 8) or to a moderate endurance training program (MET, *n* = 8). The training volume was similar in IET and MET but with different work intensities, and each lasted for 8 weeks. Participants performed a maximal graded cycle bicycle ergometer test to measure maximal oxygen consumption (VO_2_max) and maximal aerobic power (MAP) before and after the training programs. Moreover, TL_CO_, TL_NO_ and Vc were measured during a single breath maneuver. After eight weeks of training, all pulmonary parameters with the exception of alveolar volume (VA) and inspiratory volume (VI) (0.104 < *p* < 0889; 0.001 < ES < 0.091), measured at rest and at the end of maximal exercise, showed significant group × time interactions (*p* < 0.05, 0.2 < ES < 4.0). Post hoc analyses revealed significant pre-to-post decreases for maximal heart rates (*p* < 0.0001, ES = 3.1) and improvements for VO_2_max (*p* = 0.006, ES = 2.22) in the IET group. Moreover, post hoc analyses revealed significant pre-to-post improvements in the IET for DM, TL_NO_, TL_CO_ and Vc (0.001 < *p* < 0.0022; 2.68 < ES < 6.45). In addition, there were increases in Vc at rest, VO_2_max, TL_NO_ and DM in the IET but not in the MET participants after eight weeks of training with varying exercise intensities. Our findings suggest that the intensity of training may represent the most important factor in increasing pulmonary vascular function in young athletes.

## 1. Introduction

Pulmonary diffusing (or transfer) capacity for carbon monoxide (TL_CO_) from the alveoli to blood is used to determine the function of the alveolar–capillary membrane [1]. Measurements of TL_CO_ can predict arterial oxygen desaturation [2] and evaluate the prognosis of conditions such as emphysema and lung resection surgery [3]. The diffusing capacity of lungs is determined by the alveolar–capillary membrane diffusing capacity (DM_CO_) and pulmonary capillary blood volume (Vc) [4]. Exercise improves TL_CO_ in children [5] and adults [6] due to increases in Vc and DMco [7,8]. Running increases ventilatory performance in children [9], suggesting that intermittent exercise enhances respiratory demand [10] and that endurance activity alters the properties of the lung alveolar–capillary membrane by improving alveolar membrane conductance [11]. However, the magnitude of training-related cardiopulmonary adaptation depends on both the intensity and duration of the exercise training programs [6]. The specificity of the training stimulus is related to the exercise modality used (endurance, strength or speed). Several studies report improvements in TL_CO_ following chronic exercise training in healthy adults [12]. Furthermore, it is generally accepted that high-intensity endurance training improves pulmonary function more than low-intensity endurance training [13]. 

In addition, linear relationships exist between DM_CO_ measured at rest, aerobic capacity [14] and exercise performance in adults [15]. These findings suggest that improvements in endurance performance (or aerobic capacity) should also improve TL_CO_. The effects of an intense aerobic training program on pulmonary diffusing capacity (TL) at rest and during maximal exercise in young athletes are unknown. Moreover, the mechanisms by which intense endurance training, compared to a moderate endurance training program, improves pulmonary diffusion capacity in young athletes are speculative. In fact, several studies involving young participants suggest that such effects are due either to improvements in the pulmonary exchange surface or to better vascularization of the pulmonary capillary bed [11,16,17]. 

The aim of this study was to investigate the effects of an intense endurance training program compared to a moderate endurance training program on the DM_CO_ and Vc during an eight-week study in young athletic males. We hypothesized that (i) an intense endurance training program, compared to the moderate endurance training one, could produce greater increases in TL_CO_, Vc and DMco and (ii) increases in these parameters (TL, Vc and DM) may be associated with increased pulmonary vascular development and lead to greater distensibility of the pulmonary circulation in young athletes.

## 2. Materials and Methods

### 2.1. Participants

**Figure 1:**
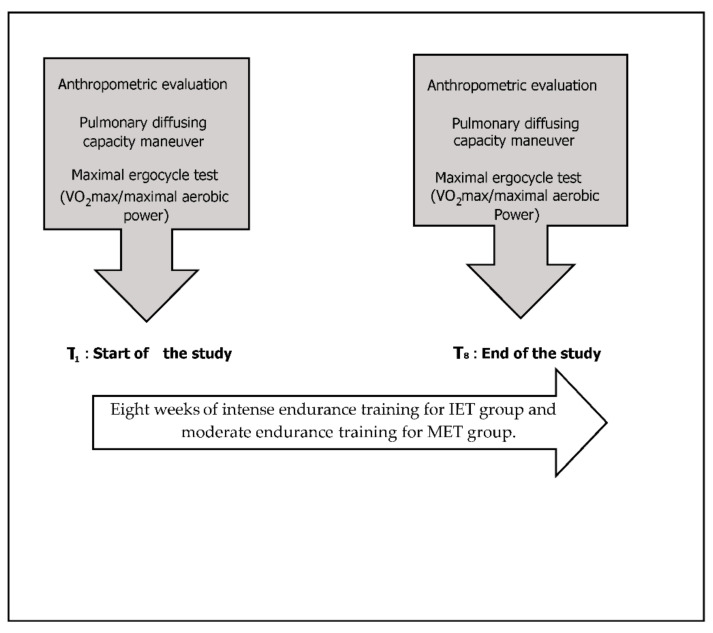
Experimental design.

Sixteen healthy young male athletes (16–18 years old) participated in the study and were randomly assigned to two different training groups: an intense endurance training program (IET, *n* = 8) and a moderate training program (MET, *n* = 8). The participants were middle-distance runners recruited from an athletic center in Nabeul (Tunisia). All participants had been engaged in systematic training programs and in national competitions during the previous six years. The participants were non-smokers with normal vital capacities and no histories of cardiopulmonary diseases or allergies. A schematic representation of the experimental design is illustrated in Figure 1. 

An a priori power analysis (expected SD of residuals, desired power = 0.90 and alpha error = 0.01) was computed using GPower 3.1 software (Version 3.1, University of Dusseldorf, Germany) to simulate a statistically significant group-by-time interaction for TL, our primary outcome [18]. The analysis indicated that a total sample size of 16 would be sufficient to achieve medium-sized group-by-time interaction effects. Written informed consent for participation was obtained from each subject and their parents or guardians prior to the study after receiving verbal and written explanations on the risks and benefits of the experimental protocol. The ethics committee of the Sousse Medical University (Tunisia) approved the study, which was in accordance with the latest version of the Declaration of Helsinki. The physical characteristics of the study participants at the time of inclusion are listed in Table 1. There was no difference between groups for any of these parameters at baseline.

### 2.2. Procedures

Baseline (T1) anthropometric data (height to the nearest 0.1cm and weight to the nearest 100 g) were collected using standard stadiometers (Seca™, Hamburg, Germany) and scales (Tefal, France). Maximal oxygen consumption (VO_2_max) and maximal aerobic power (MAP) were determined using standard protocols with exercise performed on a bicycle ergometer (Monark cycle). The subjects performed unloaded cycling at 60–65 revolutions/min (rpm) for the first minute after which the work rate was increased every minute according to the Cooper and Weiler-Ravell procedure until VO_2_max was reached [19]. Oxygen consumption (VO_2_) and carbon dioxide (VCO_2_) production were determined using a calibrated metabolic measurement system (MedGraphics CPX St Paul, MN, USA). 

The transfers of nitric monoxide (NO) and carbon monoxide (CO) were measured on the same day. Each participant performed three validated transfer measurements: two at rest (before exercise) and another at the end of maximal exercise. Exercise was performed similarly to the last standard protocol but with incremental increases in workload. The validity of the maneuver for the transfer measurement was first checked by the participant performing the maneuver without hesitation, with his mouth tightly closed around the mouthpiece, and holding his breath steadily during the pre-set time. The validity was then checked by examining the trace depicting volume changes during the maneuver, i.e., the computer-generated trace should lack a pause during the fast inspiration, be flat during the breath hold and be continuous during expiration. The results were considered valid if these criteria were met. All subjects were trained previously in these maneuvers. Transfers of NO (TL_NO_) and CO (TL_CO_) measurements were realized simultaneously during a single breath maneuver using an automated apparatus (Medisoft, Dinant, Namur, Belgium) following the latest ERS Guidelines [20]. 

DM and Vc values were determined from TL_NO_ and TL_CO_ values as previously described [21]. Since the reactivity of NO and hemoglobin was considered very high and its inverse negligible, TL_NO_ was considered equivalent to DM_NO_. DM_CO_ was determined using the coefficient of proportionality (a) and the DM values of the two gases (aDM_NO_ = aDM_CO_ = 1.97) following Graham’s law. The reactivity of CO with hemoglobin at a PO2 of 110 mmHg was derived from the relationship published by Forster [22] in which measurements were carried out at physiological pH values [23]. No corrections were made for hemoglobin concentrations as Stam et al. [24] reported that corrections have a limited effect on TL_NO_, TL_CO_ and DM values in healthy individuals [25].

The same tests (maximal oxygen consumption, maximal aerobic power and NO/CO transfer) were repeated 8 weeks later (T2). All resting tests and exercise measurements were performed using the same equipment, calibrated using identical methods and measured with identical laboratory techniques during the initial (T1) and follow-up (T2) tests. Moreover, to minimize any effects of diurnal variation, the two testing sessions were conducted within 2 h at the same time of the day (Figure 1).

Changes in alveolar volume (VA), pulmonary diffusing capacity for CO (TL_CO_) and NO (TL_NO_), membrane factor for CO (DM_CO_), lung capillary blood volume (Vc), inspiratory volume (VI) and residual volume (VR) were measured in resting subjects before (T1) and after the training programs (T2) and also immediately after maximal exercise in the IET (following intense endurance training program) and MET (following moderate endurance training protocol) groups.

### 2.3. Exercise Training Program

Experienced coaches and sports scientists trained the IET and MET groups during the eight-week intervention period. Only the IET group followed a maximal training program while the MET group followed a moderate training program (Table 2). All athletes were free of injuries during the training and testing periods. Each training session was supervised, and heart rates were measured with portable heart rate monitors (Sport-Tester PE4000). Subjects did not participate in any other physical training activities during the study. Details of the training programs are summarized in Table 2. Participants exercised 3 days per week (Monday, Thursday and Saturday) during weeks 1 to 4 of the intervention and exercised 4 days per week (Monday, Tuesday, Thursday and Friday) from week 5 to 8. Each exercise session lasted for 90 min and started and ended with a 10 min stretching period. The sessions included running distances of 400 m, 600 m, 800 m and 1500 m that were separated by active recovery periods. All training sessions were performed on an athletic track (400 m). Training volumes (running distances) were identical in the two groups, but the intensities were different and determined according to maximum heart rate (HRmax): low intensity ≈60% of HRmax, moderate endurance training 70–80% of HRmax and intense endurance training 85–95% HRmax [26,27]. Training sessions were performed during the afternoon (3:30 to 5:00 p.m.). Athletes received detailed instructions on performing each series of protocols and were always supervised. 

The athletes in both groups completed all aspects of the training programs, with nobody experiencing any injuries related to training or testing during the experimental period. The attendance rate during the 8-week training period and for rest and exercise measurements was 96%.

### 2.4. Statistical Analyses

All results are presented as means and standard deviations (SDs). After normality of data distribution was confirmed using the Shapiro–Wilk test, differences within and between groups were calculated using a two-way analysis of variance (ANOVA) for repeated measures. A Bonferroni post hoc test was calculated if group × time interactions were significant. Effect sizes (ESs) were determined from ANOVA output by converting partial eta squared to Cohen’s d values [28]. Moreover, within-group ESs were computed using the equation: ES = (mean post-mean pre)/pooled SD, and were considered trivial (<0.2), small (0.2–0.6), moderate (0.6–1.2), large (1.2–2.0) and very large (2.0–4.0). The level of significance was set at *p* < 0.05. All statistical analyses were computed using SPSS for Windows, version 16.0 (SPSS Inc., Chicago, IL, USA).

## 3. Results

Our results have a high test–retest reliability, with interclass correlation coefficients (ICCs) of 0.92 for TLco and an ICC of 0.87 for Vc (Table 3). These ICC values were near ideal according to the classification of Landis and Koch [29]. 

Changes in pulmonary and functional parameters are shown in Table 4 and Table 5.

### 3.1. Time-Related Effects

#### 3.1.1. At Rest

Changes in resting heart rate (HR) were not significantly different after the training period for either group: from 63.50 ± 1.77 to 62.00 ± 1.60 beats/min (IET) and from 64.50 ± 2.33 to 63.75 ± 0.89 (MET), but HRmax decreased only for the IET group (from 196.75 ± 2.12 to 189.70 ± 1.28 beats/min, *p* < 0.001). Time-related effects were greatest for resting HR values (*p* = 0.09, ES = 0.10) (Table 4).

Most pulmonary parameters at rest led to significant time effects (post-test > pre-test, *p* < 0.05), except for VA (*p* = 0.72, ES = 0.005) and VI (*p* = 0.21, ES = 0.05) (Table 4). Magnitudes of ESs ranged from small to large for all parameters (0.2 < ES < 4.0).

#### 3.1.2. After Maximal Exercise

HRmax and VO_2_max were affected by time (*p* = 0.003, ES = 0.28; *p* = 0.003, ES = 0.28 respectively). Most parameters measured at the end of maximal exercise showed significant effects of time (post-test > pre-test, *p* < 0.05), except for VA (*p* = 0.068, ES = 0.0114) and VI (*p* = 0.553, ES = 0.013). The magnitudes of ESs ranged from small to large for all parameters (0.2 < ES < 4.0).

### 3.2. Group Effects

#### 3.2.1. At Rest

Resting HR and most other parameters measured at rest showed group effects (*p* < 0.05) except for VA, VI and DM. Magnitudes of ESs ranged from small to large for all parameters (0.2 < ES < 4.0).

#### 3.2.2. After Maximal Exercise

HRmax and VO2max showed significant group effects (*p* < 0.0001, ES = 0.36; *p* = 0.03, ES = 0.15, respectively). Most other parameters measured at the end of maximal exercise also showed significant group effects (*p* < 0.05), except for VA and VI. Magnitudes of ESs ranged from small to large for all parameters (0.2 < ES < 4.0).

### 3.3. Group × Time Interactions

#### 3.3.1. At Rest

Most pulmonary parameters measured at rest showed significant group × time interactions (*p* < 0.05), except for VA and VI. Magnitudes of ESs ranged from small to large for all parameters (0.2 < ES < 4.0).

Post hoc tests for IET revealed significant pre-to-post improvements for DM (*p* < 0.0001, ES = 0.374), TL_NO_ (*p* < 0.0001, ES = 0.388), TL_CO_ (*p* < 0.0001, ES = 0.404) and VC (*p* = 0.009, ES = 0.222).

#### 3.3.2. After Maximal Exercise

An intense endurance training program intervention led to significant group × time interactions for HRmax (*p* < 0.0001, ES = 0.52) and VO_2_max (*p* = 0.006, ES = 0.24). Post hoc analysis revealed significant pre-to-post decreases for HRmax (*p* < 0.0001, ES = 3.1) and improvements for VO_2_max (*p* = 0.006, ES = 2.22) in the IET group.

Most pulmonary parameters measured at the end of maximal exercise showed significant group × time interactions (*p* < 0.05), except for VA (*p* = 0.104, ES = 0.091) and VI (*p* = 0.889, ES = 0.001). ES magnitudes ranged from small to large for all parameters (0.2 < ES < 4.0). Post hoc analysis revealed significant pre-to-post improvements in the IET for DM (*p* = 0.009, ES = 3.69), TL_NO_ (*p* = 0.002, ES = 2.68), TL_CO_ (*p* = 0.022, ES = 6.45) and Vc (*p* < 0.001, ES = 3.94).

## 4. Discussion

We examined the impact of eight weeks of a varying aerobic training program on pulmonary diffusing capacity, alveolar capillary membrane diffusing capacity and capillary pulmonary blood volume in two groups of young athletes using protocols having the same volumes but different intensities. The key findings were: (1) VO_2_max increased by approximately 5% (*p* = 0.003) in the IET group but not in the MET group. (2) The initial measures (T_1_) of TL_NO_ and DM were similar in the IET and MET both at rest and after maximal exercise but were increased by exercise in both groups; i.e., there was an increase (8%, *p* < 0.01) following 8 weeks of intense endurance training (IET). (3) Vc in IET was lower than in MET at rest, although Vc was not different after exercise; i.e., exercise increased Vc at rest in the IET group but not in the MET group. Thus, these results validate our hypothesis that an intense endurance training program, compared to moderate endurance training, produces greater increases in TL, Vc and DM. The improvements of these pulmonary parameters could be due to an increase in alveolar growth or an increase in permeability of the alveolar–capillary membrane, which can consequently generate a more distensible pulmonary circulation in young athletes [4]. Thus, intense exercise can lead to a high ventilation rate which can induce mechanical stress between the lung and thorax such that alveolar growth could theoretically occur [30]. At the same time, the rise in ventilation and pulmonary blood flow due to exercise can increase angiogenic growth factors [31] which could adjust capillary permeability and support the integrity of the alveolar–capillary barrier [32]. 

### 4.1. Effects of Endurance Training on Performance

Previous studies reported that increased physical activity improves maximal aerobic function [11,18]. Our study demonstrates that endurance training increased VO_2_max by 5% only in the IET but not in the MET group, indicating that a moderate endurance training program is sufficient to maintain VO_2_max but not to improve it. The slight increase in VO_2_max associated with the small decrease in HRmax can be attributed partly to hypervolemia induced by increases in cardiac stroke volume due to the training [33,34,35,36,37]. Another study [38] suggested that improvement in VO_2_max after endurance training was attributed to increases in cardiac output. In our study, the increase of VO_2_max in the IET group could be explained by the intensity of the endurance training invoking greater adaptive response.

### 4.2. Effects of Endurance Training on Lung Function

There remains uncertainty about whether high-intensity aerobic interval training can provide physiological benefits similar to those seen in continuous, moderate-intensity exercise training and particularly if pulmonary diffusing capacity can be augmented by high-intensity aerobic training in healthy humans [4]. Regardless of the mechanism by which pulmonary transfer capacity enhances, there is little research on how various types of physical exercise can influence pulmonary diffusing capacity. Our study indicates that endurance exercise training improves pulmonary function. That is, Vc in the IET group was increased by 5% at rest, but during exercise, it increased slightly but significantly by 1.5%. The increase in total lung capacity at rest may be explained by our results on changes in VA (Table 4 and Table 5). In fact, during exercise, both Vc and residual volumes increased slightly after training. Other studies show that pulmonary diffusing capacity is not altered with training. Five months of training failed to increase TL_CO_ measured at rest or during exercise [39]. Furthermore, intense endurance training increased DM by 6% (*p* = 0.001), likely due to a 6% increase in lung volume. In accordance with our study, other authors also report improvements in pulmonary diffusing capacity with chronic exercise training in adults [12,40], although some studies observed no changes [15,41]. The pulmonary diffusing capacity was not altered with training after five months of training, and there were no increases in TL_CO_ measured at rest or during exercise [39]. The relationship between lung volume and DM has been widely studied both on an experimental and theoretical basis [24,42]. 

Several studies demonstrated that exercise increased Vc in adults [8,43] and also in children [11]. Our findings indicate that endurance exercise significantly increased Vc values both at rest and after exercise training. However, diffusing capacity is influenced by Vc and DM, which is a function of lung volume. Vc measurements can also be influenced by VA [7,11,44,45], and thus the 6% increase in VA we found in the IET group is unlikely to explain the 13% increase in Vc. With incremental exercise, TL_CO_ should increase to satisfy the increased oxygen consumption; if not, a diffusion limitation may occur. The increment in TL_CO_ encountered with exercise is due to increased Vc and DM. Both Vc and DM increased secondary to the recruitment and distension of pulmonary capillaries, expanding the surface area for gas exchange [46] and declining pulmonary vascular resistance, thus attenuating the increase in pulmonary capillary pressure. A key determinant of Vc is the pulmonary capillary pressure [47], which depends on pulmonary arterial pressure, which in healthy subjects, is correlated with cardiac blood flow [6,48]. An increase in cardiac blood flow may have contributed to the increase in Vc we observed. Increases in cardiac blood flow due to endurance exercise are mainly due to changes in heart rate in healthy subjects [16], although this is unlikely to explain our findings as maximal heart rates did not differ before and after training either in the IET or MET group. 

Lung capillaries are not all perfused at high vascular pressures, and the distribution of lung capillaries shows great experimental variability [49]; intensive endurance training stimulates cardiac blood flow (Q’c) and increases pulmonary arterial pressure and pulmonary capillary pressure which would induce an increase in Vc by recruitment and distension of capillaries. This type of in vivo analysis in healthy humans is impossible as Pcap can be measured only invasively and no method to estimate the distribution of blood flow at microscopic levels currently exists. 

The 16% increase in the maximal workload was associated with a smaller 5% increase in VO_2_max, indicating that muscles were possibly more efficient in extracting oxygen [38,39,40], likely due to better distribution of blood flow in the capillaries [11,43,50]. In this context, our study is in accordance with several studies that established associations between VO_2_max and both TL_CO_ [51,52] and pulmonary hemodynamics [51,53].

### 4.3. Limitations of the Study

The present study has a number of limitations that warrant discussion. First, we examined only one type of athletic team. Hence, our sample size and the training regime of the subjects may not be optimal or generalizable; clearly, a larger subject group would have helped to better explain the relationship between endurance training intensity and lung diffusion capacity. Second, only a limited number of physiological tests were applied in our evaluations. Thus, our findings are specific to these physiological outcomes only. 

## 5. Conclusions

The main findings of the present study indicate that (A) an intense endurance exercise in young athletes improves pulmonary vascular function and (B) increases in DM and Vc at rest and during exercise are likely due to greater recruitment of lung capillaries. Although the exact mechanisms by which endurance training improves lung diffusion are not well defined, it is likely that increases in the total surface area of the lungs for gas exchange and/or by alterations in alveolar/vascular membrane thickness play an important role. It is possible that the plasticity of the pulmonary vascular bed in young athletes could contribute to improvements in lung perfusion during exercise. 

## Figures and Tables

**Table 1:** Subject characteristics and maximal exercise performances of athletes at the start of the study (T1). Data are mean ± standard deviation (SD). IET group: intense endurance training group; MET: moderate endurance training group.

	IET Group (n = 8)		MET Group *p*-Value (n = 8)
Age (years)	17.0 ± 1.0		17.1 ± 0.6 0.76
Weight (kg)	64 ± 2		65 ± 2 0.46
Height (cm)	172 ± 7		172 ± 4 1
VO_2_max (mL·kg^−1^ min^−1^)	47.2 ± 1.4		47.5 ± 1.2 0.67
Maximum Work Load (w)	245 ± 30		250 ± 37 0.77
Heart Rate (beats/min)	rest	63 ± 2	64 ± 2 0.35
	max	197 ± 2	196 ± 2 0.32

**Table 2:** Training programs for intense endurance training group (IET) and moderate endurance training group (MET).

	Intense Endurance Training Group (IET)	Moderate Endurance Training Group (MET)
Weeks 1 to 4	Weekly Training Program	
Monday	Warm-up/Drills: 30 min Running 600 m/800 m/1000 m/1500 m: moderate intensity 70–80% of HRmax Active recovery = 2–3 min	Warm-up/Drills: 30 min Running 600 m/800 m/1000 m/1500 m: low intensity (aerobic exercises) ≈60% of HRmax Active recovery = 2–3 min
Tuesday	Recovery	Recovery
Wednesday	Recovery	Recovery
Thursday	Warm-up/Drills: 30 min Running 400 m/600 m/800 m: heavy endurance training 85–95% of HRmax Active recovery = 2–3 min	Warm-up/Drills: 30 minRunning 400 m/600 m/800 m: moderate endurance training 70–80% of HRmaxActive recovery = 2–3 min
Friday	Recovery	Recovery
Saturday	Warm-up/Drills: 30 min Running 600 m/800 m/1000 m/1500 m: moderate intensity 70–80% of HRmax Active recovery = 2–3 min	Warm-up/Drills: 30 min Running 600 m/800 m/1000 m/1500 m: low to moderate intensity ≈60–70 of HRmax Active recovery = 2–3 min
Sunday	Recovery	Recovery
Weeks 5 to 8	Weekly Training Program	
Monday	Warm-up/Drills: 30 min Running 400 m/600 m/800 m: moderate to heavy endurance training 70–95% of HRmax	Warm-up/Drills: 30 min Running 400 m/600 m/800 m: low intensity ≈ 60% of HRmax
Tuesday	Warm-up/Drills: 30 min Running 600 m/800 m/1000 m/1500 m): moderate endurance training 70–80% of HRmax Active recovery = 2–3 min	Warm-up/Drills: 30 min Running 600 m/800 m/1000 m/1500 m: low endurance training ≈ 60% of HRmax Active recovery = 2–3 min
Wednesday	Recovery	Recovery
Thursday	Warm-up/Drills: 30 min Running 800 m/1000 m/1500 m: 85–95% of HRmax Active recovery = 2–3 min	Warm-up/Drills: 30 min Running 800 m/1000 m/1500 m: 70–80% of HRmax Active recovery = 2–3 min
Friday	Warm-up/Drills: 30 min Running 600 m/800 m/1000 m/1500 m: moderate to heavy intensity 70–95% of HRmax Active recovery = 2–3 min	Warm-up/Drills: 30 min Running 600 m/800 m/1000 m/1500 m: low intensity ≈ 60% of HRmax Active recovery = 2–3 min
Saturday	Recovery	Recovery
Sunday	Recovery	Recovery

**Table 3:** Intraclass correlation coefficients (ICCs) for relative reliability and coefficients of variation for absolute reliability. Data are mean ± standard deviation (SD) of VI: inspiratory volume; VA: alveolar volume; TL_NO_: pulmonary diffusing capacity for nitric oxide; DL_CO_: pulmonary diffusing capacity for carbon monoxide; Dm: membrane component of alveolar–capillary transfer of gases; Vc: capillary blood volume. IET group: intense endurance training group; MET: moderate endurance training group.

		Mean ± SD	*p* (Paired *t* Test)	ICC	ICC: CI 95%	CV (%)
VI (L)						
Rest	1st trial	5.18 ± 0.18	0.48	0.92	0.78–0.97	1.73
	2nd trial	5.17 ± 0.15				
Exercise	1st trial	5.45 ± 0.24	0.07	0.89	0.68–0.96	2.65
	2nd trial	5.38 ± 0.22				
VA (L)						
Rest	1st trial	7.48 ± 0.33	0.14	0.89	0.69–0.96	2.63
	2nd trial	7.40 ± 0.29				
Exercise	1st trial	7.56 ± 0.35	0.69	0.96	0.89–0.99	1.76
	2nd trial	7.54 ± 0.34				
DLNO (mL·min^−1^·mmHg^−1^)						
Rest	1st trial	196.78 ± 1.32	0.13	0.80	0.41–0.93	1.62
	2nd trial	196.29 ± 1.63				
Exercise	1st trial	256.46 ± 5.97	0.08	0.91	0.75–0.97	1.34
	2nd trial	254.28 ± 6.07				
DLCO (mL·min^−1^·mmHg^−1^)						
Rest	1st trial	47.84 ± 0.79	0.71	0.84	0.54–0.94	1.23
	2nd trial	47.46 ± 0.58				
Exercise	1st trial	53.92 ± 2.41	0.92	0.94	0.83–0.98	2.07
	2nd trial	53.89 ± 2.32				
DM (mL·min^−1^·mmHg^−1^)						
Rest	1st trial	97.43 ± 2.84	0.56	0.96	0.86–0.98	1.24
	2nd trial	97.25 ± 3.05				
Exercise	1st trial	131.35 ± 2.57	0.11	0.96	0.89–0.99	0.81
	2nd trial	130.89 ± 2.96				
VC (mL)						
Rest	1st trial	130.79 ± 6.68	0.38	0.94	0.84–0.98	2.38
	2nd trial	131.50 ± 6.92				
Exercise	1st trial	159.36 ± 1.24	0.87	0.82	0.48–0.94	0.86
	2nd trial	159.41 ± 2.14				

**Table 4:** Pulmonary parameters measured at rest: alveolar volume (VA), nitric oxide lung transfer (TL_NO_), carbon monoxide lung transfer (TL_CO_), membrane factor for CO (Dm_CO_), lung capillary blood volume (Vc), inspiratory volume (VI), residual volume (VR) and resting heart rate (HRr) before and after training program in experimental and control groups. Values for interaction effects and effect sizes (ESs) are also shown. Data are mean ± standard deviation (SD) of alveolar volume (VA), nitric oxide lung transfer (TL_NO_), carbon monoxide lung transfer (TL_CO_), membrane factor for CO (Dm_CO_), lung capillary blood volume (Vc), inspiratory volume (VI), residual volume (VR) and resting heart rate (Resting HR), intense endurance training (IET) and moderate endurance training (MET).

Parameter	IET Group (Mean ± SD)			MET Group (Mean ± SD)			Variance Analysis/Effects					
							Group		Time		Group × Time	
	Pre	Post	ES	Pre	Post	ES	*p*	ES	*p*	ES	*p*	ES
VI (L)	5.17 ± 0.22	5.43 ± 0.32	0.94	5.26 ± 0.27	5.22 ± 0.08	0.2	0.526	0.015	0.214	0.055	0.091	0.099
DM (mL/min/mmHg)	96.06 ± 3.90	108.85 ± 3.69	3.36	99.69 ± 2.87	101.84 ± 4.11	0.61	0.203	0.057	0.000	0.541	0.000	0.374
TLNO (mL/min/mmHg)	189.21 ± 3.63	213.16 ± 8.98	2.72	197.08 ± 1.83	200.24 ± 2.62	1.4	0.003	0.279	0.000	0.567	0.000	0.388
TLCO (mL/min/mmHg)	46.88 ± 1.46	53.43 ± 3.18	2.36	47.89 ± 3.93	47.84 ± 1.23	0.02	0.000	0.411	0.000	0.407	0.000	0.404
Vc (mL)	131.68 ± 10.17	150.05 ± 3.39	2.42	130.96 ± 7.75	130.51 ± 14.51	0.08	0.009	0.221	0.016	0.190	0.009	0.222
VA (L)	7.40 ± 0.04	7.52 ± 0.15	1.09	7.46 ± 0.37	7.42 ± 0.45	0.1	0.857	0.001	0.724	0.005	0.449	0.021
Resting HR (bpm)	63.5 ± 1.7	62 ± 1.8	0.84	64.5 ± 2.3	63.8 ± 0.9	0.35	0.03	0.16	0.09	0.10	0.48	0.09

**Table 5:** Comparison between the intense endurance training group (IET) and moderate endurance training group (MET) before and after 8-week period. Values for interaction effects and effect sizes (ESs) are also shown. Data are mean ± standard deviation (SD) of alveolar volume (VA), nitric oxide lung transfer (TL_NO_), carbon monoxide lung transfer (TL_CO_), membrane factor for CO (Dm_CO_), lung capillary blood volume (Vc), inspiratory volume (VI), residual volume (VR), maximal heart rate (HRmax), maximal oxygen uptake (VO_2_max), intense endurance training (IET) and moderate endurance training (MET).

Parameter	IET Group (Mean ± SD)			MET Group (Mean ± SD)			Variance Analysis/Effects					
							Group		Time		Group × Time	
	Pre	Post	ES	Pre	Post	ES	*p*	ES	*p*	ES	*p*	ES
VI (L)	5.41 ± 0.43	5.49 ± 0.22	0.23	5.36 ± 0.29	5.41 ± 0.2	0.2	0.537	0.014	0.553	0.013	0.889	0.001
DM (mL/min/mmHg)	131.3 ± 3.21	140.09 ± 1.00	3.69	131.4 ± 1.96	132.56 ± 6.63	0.24	0.011	0.211	0.001	0.324	0.009	0.220
TLNO (mL/min/mmHg)	256.39 ± 8.59	276.91 ± 6.59	2.68	256.54 ± 1.59	261.04 ± 7.79	8	0.003	0.281	0.000	0.498	0.002	0.289
TLCO (mL/min/mmHg)	53.71 ± 0.85	59.03 ± 0.80	6.45	54.14 ± 3.41	54 ± 5.21	0.03	0.049	0.131	0.028	0.160	0.022	0.174
Vc (mL)	159.09 ± 0.63	179.31 ± 7.23	3.94	159.15 ± 0.58	160.48 ± 8.81	0.21	0.000	0.435	0.000	0.504	0.000	0.439
VA (L)	7.49 ± 0.38	7.96 ± 0.30	1.37	7.50 ± 0.27	7.53 ± 0.47	0.05	0.125	0.082	0.068	0.114	0.104	0.091
HRmax (bpm)	196.7 ± 2.1	189.7 ± 1.2	3.1	195.7 ± 1.7	196.1 ± 2.3	0.19	0.000	0.36	0.000	0.46	0.000	0.52
VO_2_max (mL/kg/min)	47.2 ± 1.39	49.7 ± 0.66	2.22	47.5 ± 1.23	47.7 ± 0.91	0.12	0.03	0.15	0.003	0.28	0.006	0.24

## Data Availability

The datasets generated for this study are available on request to the corresponding author.
